# Effect of apparent metabolizable energy and sex on predictive models for growth performance, carcass traits, gut characteristics, and pellet quality in broiler chicks

**DOI:** 10.1016/j.psj.2025.106094

**Published:** 2025-11-10

**Authors:** Mohammad Hossein Mohammadi, Mahya Pahlavan Hassan, Enayat Rahmatnejad

**Affiliations:** a Department of Animal Science, College of Agriculture and Natural Resources, University of Tehran, P. O. Box, 31585-4111 Karaj, Iran; b Department of Animal Science, Faculty of Agriculture and Natural Resources, Persian Gulf University, Bushehr 75169, Iran

**Keywords:** AMEn, Broiler, Prediction equations, Performance, Sex

## Abstract

This study aimed to investigate the effects of different dietary apparent metabolizable energy (AMEn) levels and bird sex on prediction models for growth performance, carcass traits, gut characteristics, and physical pellet quality (PPQ) of starter and finisher diets in broiler chicks over a 43-d period. A total of 700 Hubbard Flex feather-sexed broiler chicks were randomly assigned to 10 treatment groups (five replicates with 14 birds/replicate) within a completely randomized design across three feeding phases: starter (1 to 14 days), grower (15 to 28 days), and finisher (29 to 43 days). Diets were arranged factorially (5 × 2) with five levels of AMEn (2850, 2950, 3050, 3150, and 3250 kcal/kg), and two sexes (male and female). The results indicated a significant interaction between dietary AMEn and sex for growth performance, carcass traits, and gut characteristics, except for carcass yield, abdominal fat pad (AFP), and the relative weight of liver and leg (*P* < 0.05). In both sexes, birds fed diets containing 3050 kcal/kg AMEn across the starter, grower, and finisher phases showed the best growth performance from day 1 to 42 compared to other treatments (*P* < 0.05). Additionally, providing broilers with dietary AMEn levels higher than 3050 kcal/kg did not result in significant improvements in growth performance and carcass yield. Dietary AMEn significantly affected carcass yield, and sex had a significant impact on AFP at day 43 (*P* < 0.05). Birds receiving 2850 kcal/kg diets had the lowest carcass yield, whereas female broilers showed a higher AFP than males (*P* < 0.05). Dietary AMEn significantly influenced the pellet durability index (PDI) of finisher feeds (*P* < 0.05), with the 3050 kcal/kg diet exhibiting the highest PDI among all treatments (*P* < 0.05). In conclusion, increasing dietary AMEn levels beyond 3050 kcal/kg did not lead to linear improvements in growth performance, carcass traits, gut organ weights, and pellet quality in both male and female broiler chickens.

## Introduction

Poultry nutritionists prioritize proper dietary energy levels due to high feed costs (Martinez et al., 2025). The costs of dietary energy in feed formulation are significant, with lipids providing the highest caloric value among nutrients (Wang et al., 2020).

The dietary apparent metabolizable energy (AMEn) level is one of the most effective factors controlling the feed intake (FI) in birds, which is influenced by several factors (Classen, 2017; Hu et al., 2019). The provision of dietary AMEn influences nutrient intake due to balanced nutrient density (the relationship of nutrients to dietary AMEn level) in feed formulation (Lopez et al., 2008; Muñoz et al., 2018).

In general, previous studies have shown that increasing dietary AMEn levels linearly reduces FI (Saleh et al., 2004; Lemme et al., 2005; Wang et al., 2020). In contrast, reducing dietary AMEn by 100 kcal/kg decreased FI and the relative weight of the abdominal fat pad (AFP) of Cobb500 male broilers fed pellet diets (Ko et al., 2023). In a study with male and female Ross 708 broilers fed pelleted diets, increasing the dietary AMEn level from 3175 to 3310 kcal/kg resulted in a linear reduction in FI and FCR from 30 to 59 days of age, without affecting body weight (BW), AFP, carcass yield, and breast meat yield (Dozier et al., 2006). In some studies, broiler chicks fed a different range of dietary AMEn eventually had the same BW (Dozier et al., 2007; Lopez and Leeson, 2008; Sharma et al., 2018; Johnson et al., 2020).

Svihus (2006) indicated that the macrostructure of pellets, measured by pellet physical quality (PPQ) parameters, namely pellet durability index (PDI) and pellet hardness (PH), play a role on FI and BW of broilers. According to Mohammadi Ghasem Abadi et al. (2019a), PPQ parameters, which are influenced by feed formulation and particle size (60 %), as well as other feed milling process parameters (40 %), have a significant impact on broiler growth performance. It was noted that adding 1 to 4 % mixer-added fat (MAF) increases dietary AMEn density; however, it does not improve growth performance and adversely affects PPQ (Gehring et al., 2011; Dozier et al., 2011; Mohammadi Ghasem Abadi et al., 2019b).

Abdollahi et al. (2018) reported that increasing dietary AMEn levels reduced FI and FCR, but had no impact on the BW of broilers fed 2798 to 3200 kcal/kg pellet diets, resulting in compromised PDI from 91 to 72 %. On the contrary, in a study on Ross 308 as-hatched broilers fed pellet diets, increasing dietary AMEn levels from 2700 to 3100 kcal/kg (2700, 2833, 2966, and 3100 kcal/kg) had no effect on FI. However, the corresponding PDI values were 82.2, 81.7, 80.0, and 86.0 %, respectively (Cho, 2011).

Broiler`s response to dietary AMEn levels is influenced by genotype (strain), diet composition, feed physical form (mash or pellet), age, energy-protein ratio, and environmental conditions (Classen, 2017). In addition, the outcomes of earlier research demonstrated that different broiler strains respond uniquely to changes in dietary AMEn level (Delezie, 2010; Kim et al., 2012; Dozier and Gehring, 2014). Furthermore, there is a notable lack of research exploring the impact of dietary AMEn on Hubbard Flex broiler chicks, highlighting a significant gap in our understanding (Mostert, 2015; Martinez et al., 2025, Martins et al., 2016). Addressing this void could lead to improved growth performance for these chicks.

Maynard et al. (2019) noted that sex and dietary AMEn had no interaction effects on the growth performance and carcass traits of broiler. Madilindi et al. (2018) has shown female broilers had higher AFP percentages than male. Research indicates that female broilers have a reduced response to excess dietary AMEn due to their lower growth rates, leading to harmful AFP accumulation. It’s essential to optimize dietary energy levels to improve their performance (Dozier et al., 2011; Fouad et al., 2014; England et al., 2022). However, conflicting reports exist regarding the influence of dietary AMEn on carcass characteristics, especially AFP (Clark, 2015).

The study aimed to clarify how varying levels of dietary AMEn influence the growth performance, carcass characteristics, and gut development in male and female Hubbard Flex broiler chicks fed pellet diets. The findings are intended to guide poultry nutritionists in optimizing gut development and enhancing the economic viability of broiler production.

## Materials and methods

### Experimental design and bird management

All procedures in the current study were approved by the Local Experimental Animal Care Committee within the Department of Animal Science, Faculty of agriculture, Persian Gulf University, Busher, Iran.

A total of 700 one-day-old male and female Hubbard Flex chicks were reared on 50 wood-shaving floor pens (1.25×1.25 m^2^; 10 treatments with 5 replicate) at maximum stock density (14 birds/pen) in an environmentally controlled broiler house located at Gorgan, Iran. Both sexes fed five levels of dietary AMEn (2850, 2950, 3050, 3150, and 3250 kcal/kg) from 1 to 43 days of age. Temperature and light programs were settled based on the Hubbard™ broiler management guide (2016). Feed and water were provided ad libitum. Birds were vaccinated according to recommendations of a local veterinarian. In brief, chicks were sprayed with the B1+H120 vaccine at the hatchery. To achieve optimal immunity against the Newcastle disease, they received the vaccine at 8 days of age by eye and neck drop method and at 17 and 24 days of age by drinking water method. Additionally, the infectious bursal disease vaccine was also administered at 15 days of age via drinking water.

### Diets and bird performance, carcass traits and gut characteristics

Five diets with different AMEn levels (2850, 2950, 3050, 3150, and 3250 kcal/kg) with balanced nutrient density for starter (1 to 14d), grower (15 to 28d), and finisher (29 to 43d) phases were formulated based on the Hubbard™ broiler management guide (2016), as detailed in Table 1. The feeds were processed in a twin conditioner with 40 s feed retention time, 1.5 bar steam pressure, and 65 °C discharge feed temperature, and pelleted in a belt-type pellet press (5 Ton/h) with two dies (length-to-diameter ratio = 12). Pellets were cooled at ambient temperature in a counter-flow cooler for 15 min. The physical form of the feeds for each phase was as follows: starter (pellet 2 mm diameter), grower (crumble from 4 mm diameter), and finisher (pellet 4 mm diameter).

**Table 1:** Composition and nutritional content of the experimental diets (%).

Type of diet	Starter (1 to 14 d)					Grower (15 to 28 d)					Finisher (29 to 43 d)				
Treatments	2850	2950	3050	3150	3250	2850	2950	3050	3150	3250	2850	2950	3050	3150	3250
Ingredients (%)															
Corn (7.5 % CP)	39.6	34.7	29.8	25.0	20.1	39.3	34.8	30.3	25.8	21.2	33.5	32.4	28.0	23.7	19.4
Wheat (12 % CP)	20	20	20	20	20	30	30	30	30	30	40	40	40	40	40
Soybean meal (44 % CP)	34.3	36.8	39.2	41.7	44.1	26.1	28.3	30.5	32.7	34.9	20.6	22.1	24.2	26.2	28.2
Canola oil	1.3	3.5	5.8	8.1	10.4	0	2.2	4.4	6.6	8.8	0	1.1	3.3	5.5	7.7
Dicalcium phosphate	2.1	2.2	2.3	2.3	2.4	1.8	1.9	1.9	2.0	2.1	1.4	1.5	1.5	1.6	1.7
Calcium carbonate	0.85	0.86	0.87	0.88	0.88	0.85	0.86	0.90	0.91	0.92	0.95	0.97	0.98	1.00	1.01
Sodium bicarbonate	0.35	0.34	0.34	0.33	0.32	0.48	0.43	0.42	0.42	0.42	0.41	0.42	0.41	0.41	0.40
NaCl	0.18	0.20	0.21	0.23	0.25	0.08	0.09	0.11	0.13	0.14	0.05	0.07	0.08	0.10	0.11
Vitamin and mineral premix^1^	0.5	0.5	0.5	0.5	0.5	0.5	0.5	0.5	0.5	0.5	0.5	0.5	0.5	0.5	0.5
L-lysine HCl	0.26	0.25	0.24	0.23	0.21	0.30	0.28	0.27	0.26	0.25	0.34	0.34	0.33	0.32	0.31
DL-methionine	0.33	0.35	0.37	0.39	0.41	0.28	0.3	0.31	0.33	0.35	0.26	0.28	0.29	0.31	0.33
L-threonine	0.12	0.12	0.12	0.12	0.12	0.12	0.12	0.12	0.13	0.13	0.14	0.15	0.15	0.15	0.16
Anticoccidiosis	0.05	0.05	0.05	0.05	0.05	0.05	0.05	0.05	0.05	0.05	0.05	0.05	0.05	0.05	0.05
Bentonite	0.06	0.13	0.2	0.17	0.26	0.14	0.17	0.22	0.17	0.24	1.8	0.12	0.21	0.16	0.13
Chemical analysis (calculated)															
Metabolizable energy (kcal/kg)	2850	2950	3050	3150	3250	2850	2950	3050	3150	3250	2850	2950	3050	3150	3250
Crude protein (%)	21.3	22.1	22.8	23.6	24.3	18.8	19.5	20.1	20.7	21.4	17.1	17.7	18.3	18.9	19.5
Crude fiber (%) (wet chemistry)	2.8	2.5	4.2	3.3	3.7						2.4	2.0	3.5	3.3	3
Ether extract (%) (wet chemistry)	4.6	4.8	5.2	6.5	6.2						6.1	5.2	4.6	5.5	6.1
Lysine SID (%)	1.20	1.24	1.29	1.33	1.37	1.05	1.09	1.12	1.16	1.20	0.97	1.00	1.03	1.07	1.10
Methionine + cysteine SID (%)	0.90	0.93	0.966	1.0	1.03	0.80	0.83	0.85	0.88	0.91	0.75	0.77	0.80	0.82	0.85
Threonine SID (%)	0.78	0.81	0.83	0.86	0.89	0.69	0.72	0.74	0.76	0.79	0.65	0.67	0.69	0.71	0.74
Calcium (%)	0.93	0.96	1.00	1.03	1.06	0.85	0.88	0.91	0.94	0.97	0.78	0.81	0.84	0.86	0.89
Available phosphorus (%)	0.45	0.47	0.48	0.50	0.52	0.41	0.42	0.43	0.44	0.46	0.34	0.35	0.36	0.38	0.39
Sodium (%)	0.18	0.18	0.19	0.2	0.20	0.16	0.17	0.17	0.18	0.18	0.14	0.15	0.16	0.16	0.17
Chlorine (%)	0.21	0.22	0.23	0.24	0.24	0.17	0.17	0.18	0.18	0.19	0.17	0.17	0.18	0.19	0.19
DCAB^2^ (mEq/kg)	232	240	248	257	265	216	218	225	233	241	185	192	198	205	212

^1.^ The amount of vitamins and minerals per kilogram of diet respectively in the early, growing, and finishing periods for vitamin A: 15000, 12500, and 10000 international units; Cholecalciferol: 3000, 2500, and 2000 international units; Vitamin E: 70, 50, and 50 international units; Vitamin B12: 0.02, 0.01, and 0.01 mg; Folacin: 1.5, 1, and 1 mg; Niacin: 60, 40, and 40 mg; pantothenic acid, 15, 10, and 10 mg; Pyridoxine: 4, 3, and 3 mg; Riboflavin: 8, 6, and 6 mg; Thiamine: 3, 2, and 2 mg; Choline: 400, 300, and 200 mg; Antioxidant: 0.5, 0.5, and 0.5 mg; Copper (copper sulfate): 10, 10, and 10 mg; Iodine (calcium iodate): 1, 1, and 1 mg; Iron (ferrous sulfate): 20, 20, and 20 mg; Manganese (manganese oxide): 80, 80, and 80 mg; Selenium (sodium selenate): 0.2, 0.2, and 0.2 mg; and zinc (zinc oxide): 80, 80, and 80 mg.

2. DCAB; Dietary Cation-Anion balanced (Na+K-Cl).

Proximate analysis of feed`s crude fiber and ether extract were conducted according to AOAC (2000).

Birds were weighed and distributed at the beginning of the experiment to have an equal BW using digital scale (± 1 g). Feed intake and body weight were recorded weekly; however, due to the large volume of data collected in this study, the overall growth performance is presented. Feed conversion ratio (FCR) and European production efficiency factor (EPEF) were calculated on 42d following the method described by Biesek et al. (2022) using the following formula:

EPEF=(livability,%×liveweight,kg/age,d×FCR)×100

Two birds from each replicate were selected based on the average BW of each pen on day 42. Subsequently, 100 birds, after six hours of feed deprivation, were transferred to the slaughterhouse on day 43. Each bird was individually weighed before and after slaughter to calculate carcass yield. All carcass traits were weighed and presented here according to relative organ weight (% BW).

The empty weights (± 0.1 g) of excised and fat-trimmed proventriculus, gizzard, and different segments of small intestine were determined and expressed as the percentage of BW. Moreover, the relative lengths of different parts of the small intestine (cm/BW) were measured using tape measure.

### Physical pellet quality

Clean pellet samples (500 g; four replicates per diet) were challenged with a Pfost tumbling box for 10 min at 50 RPM to determine PDI using the methods described by Mohammadi Ghasem Abadi et al. (2019b). PH (kg) was measured using a Brookfield CT3 10,000 g texture analyzer (Middleborough, USA) for 10 samples of intact pellet, with a cylindrical probe number 3, which was set for the compression test (2 mm target test and 1.5 mm/s speed), according to the method described by Svihus et al. (2004).

### Statistical Analysis

The data were analyzed using Minitab 18. GLM method with a completely randomized 5 × 2 factorial treatment arrangement including five different dietary AMEn levels (3250, 3150, 3050, 2950, and 2850 kcal/kg) and two sexes (male and female). The significance level was set at *p* < 0.05, and treatment means were compared using Tukey's test. Additionally, linear and quadratic regression for each trait and prediction models of growth performance were presented for both sexes using of Minitab 18 and the graphs were generated using by Microsoft Excel 2016.

## Results

### Growth performance

**Fig. 1:**
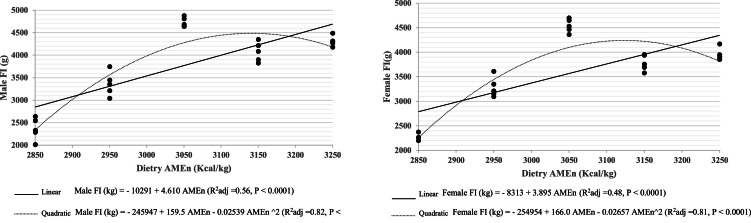
Male and female broilers' FI response to dietary AMEn (kcal/kg) from 0 to 42d.

**Fig. 2:**
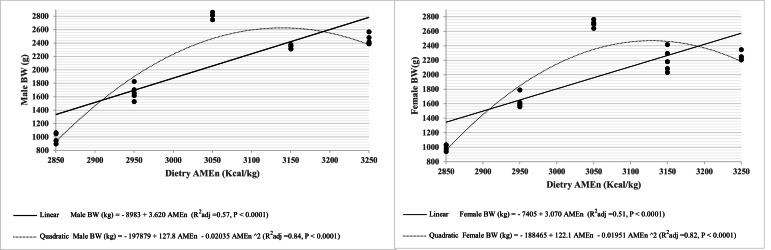
Male and female broilers' BW response to dietary AMEn (kcal/kg) at 42d.

**Fig. 3:**
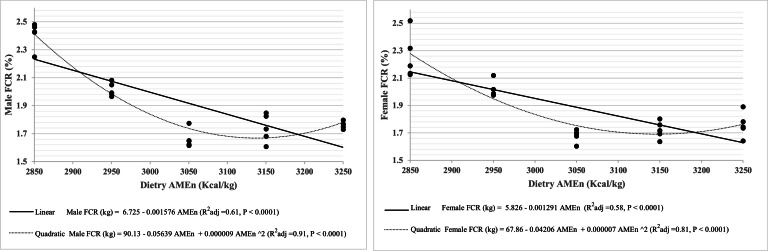
Male and female broilers' FCR response to dietary AMEn (kcal/kg) from 0 to 42d.

The effect of dietary AMEn level and bird sex on growth performance (BW, FI, FCR, and EPEF) are presented in Table 2. Additionally, FI, BW, and FCR responses to dietary AMEn with their prediction equation for both sexes have been shown in Figs. 1–3. The results of this study indicated the significant interaction of the dietary AMEn and sex on the growth performance at 42d (*P* < 0.05). At 42 days of age, chicks fed a diet containing 3050 kcal/kg had the highest BW, FI, and EPEF, along with the lowest FCR, whereas those fed 2850 kcal/kg showed the opposite trend (*P* < 0.05). Generally, in both sexes, growth performance did not increase linearly with a progressive increase in dietary AMEn level (Fig. 1, Fig. 2, Fig. 3).

**Table 2:** The effect of dietary AMEn level (kcal/kg) and sex on the growth performance of broiler chicks.

Item	AMEn	Sex	BW (g)		FI (g)	FCR	EPEF (%)
			1 d	42 d	1 to 42 d	1 to 42 d	1 to 42 d
AMEn × Sex	2850	Male	41.0	e 980	2364^f^	2.409^a^	78^d^
	2950	Male	40.6	1666^d^	3360^e^	2.015^b^	168^c^
	3050	Male	40.9	2850^a^	4734^a^	1.662^c^	344^a^
	3150	Male	40.6	2345^bc^	4076^cd^	1.739^c^	266^b^
	3250	Male	41.8	2450^b^	4311^bc^	1.759^c^	279^b^
	2850	Female	40.8	1004^e^	2261^f^	2.257^a^	86^d^
	2950	Female	40.0	1630^d^	3286^e^	2.017^b^	164^c^
	3050	Female	41.0	2706^a^	4543^ab^	1.679^c^	329^a^
	3150	Female	40.4	2202^c^	3786^d^	1.721^c^	261^b^
	3250	Female	40.2	2252^c^	3959^cd^	1.759^c^	252^b^
SEM			0.44	37.0	78.5	0.03	7.19
AMEn	2850		40.9	992^d^	2313^d^	2.333^a^	82^d^
	2950		40.3	1648^c^	3323^c^	2.016^b^	166^c^
	3050		40.9	2777^a^	4639^a^	1.670^c^	336^a^
	3150		40.5	2273^b^	3931^b^	1.730^c^	264^b^
	3250		41.0	2351^b^	4135^b^	1.759^c^	266^b^
SEM			0.31	26.1	55.4	0.02	5.07
Sex		Male	41.0	2058^a^	3769^a^	1.917	277
		Female	40.5	1959^b^	3567^b^	1.886	219
SEM			0.19	16.5	35.0	0.01	3.21
*P*-value							
AMEn			0.42	0.0001	0.0001	0.0001	0.0001
Sex			0.07	0.0001	0.0001	0.21	0.06
AMEn × Sex			0.36	0.03	0.0001	0.0001	0.0001
AMEn level							
Linear				0.0001	0.0001	0.0001	0.0001
Quadratic				0.0001	0.0001	0.0001	0.0001

a-f Means in the same row with no superscript letters or a common superscript letter after them are not significantly different (*P* > 0.05). BW; body weight, FI; feed intake, FCR; feed conversion ratio, EPEF; European production efficiency factor, n: 70 birds/treatment, SEM; standard error of the mean.

### Carcass traits

The effect of dietary AMEn level and sex on carcass traits are presented in Table 3. As can be seen, the interaction between dietary AMEn level and sex on the relative weights of breast, back, and wings, and feet was significant (*P* < 0.05). At 42 days of age, regardless of sex, broiler chicks fed diets containing 3050 kcal/kg showed the highest relative breast weight, whereas those fed 2850 kcal/kg had the lowest. In other words, in both sexes, the relative breast weight did not increase linearly with dietary AMEn levels exceeding 3050 kcal/kg.

**Table 3:** The effect of dietary AMEn level (kcal/kg) and sex on the carcass traits of broiler chicks at 43d (% of BW).

Item	AMEn	Sex	Carcass	Leg	Breast	Back	Wing	AFP	Feet
AMEn × Sex	2850	Male	70.7	28.1	20.5^e^	12.5^a^	7.8^a^	1.45	6.0^a^
	2950	Male	68.4	27.8	24.7^cd^	10.2^b^	7.0^bc^	1.39	5.0^b^
	3050	Male	74.8	28.0	28.7^ab^	10.5^b^	6.5^c^	1.27	4.0^c^
	3150	Male	74.0	29.0	26.5^bc^	10.4^b^	6.9^bc^	1.19	3.5^cd^
	3250	Male	74.4	27.7	28.6^ab^	10.0^b^	6.5^c^	1.24	3.9^cd^
	2850	Female	69.0	27.6	21.8^de^	11.0^ab^	7.4^ab^	1.97	5.2^b^
	2950	Female	72.8	28.3	25.2^c^	10.9^b^	7.0^bc^	2.00	4.0^c^
	3050	Female	75.3	28.0	30.6^a^	10.4^b^	6.3^c^	1.21	3.1^d^
	3150	Female	75.3	28.1	27.8abc	10.9^ab^	6.8^bc^	1.62	3.6^cd^
	3250	Female	74.8	26.3	30.2^a^	10.2^b^	6.6^bc^	2.02	3.4^cd^
SEM			1.58	0.58	0.69	0.33	0.17	0.21	0.16
AMEn	2850		69.8^b^	27.8	21.1^d^	11.8^a^	7.6^a^	1.71	5.6^a^
	2950		70.6^ab^	28.0	25.0^c^	10.6^b^	7.0^b^	1.69	4.5^b^
	3050		75.1^a^	28.0	29.6^a^	10.5^b^	6.4^c^	1.24	3.6^c^
	3150		74.1^a^	28.6	27.2^c^	10.7^b^	6.8^bc^	1.41	3.6^c^
	3250		74.6^a^	27.0	29.4^a^	10.1^b^	6.5^bc^	1.63	3.6^c^
SEM			1.12	0.41	0.48	0.23	0.12	0.14	0.12
Sex		Male	72.5	28.1	25.8^b^	10.7	6.9	1.31^b^	4.51^a^
		Female	73.5	10.7	27.1 a	10.7	6.8	1.77 a	3.89 b
SEM			0.70	0.26	0.30	0.15	0.07	0.09	0.08
*P*-value									
AMEn			0.002	0.13	0.0001	0.0001	0.0001	0.13	0.0001
Sex			0.33	0.22	0.004	0.86	0.28	0.002	0.0001
AMEn × Sex			0.42	0.56	0.0001	0.02	0.0001	0.36	0.03
AMEn level									
Linear			0.0001	0.40	0.0001	0.001	0.0001	0.42	0.0001
Quadratic			0.001	0.13	0.0001	0.001	0.0001	0.16	0.0001

a-e Means in the same row with no superscript letters or a common superscript letter after them are not significantly different (*P* > 0.05). AFP; abdominal fat pad, n: 10 birds/treatment, SEM; standard error of the mean.

The study observed that both male and female chicks on a diet of 2850 kcal/kg exhibited the highest relative weights of back, wings, and feet, indicating a linear and quadratic relationship between these weights and increasing dietary AMEn levels.

The legs’ relative weight remained unchanged across different AMEn levels, sex, or their interaction. In other words, there were no linear and quadratic relationships between leg relative weight and AFP percentage and increasing dietary AMEn levels.

Carcass yield showed significant variation (*P* = 0.002), with chicks on the lowest diet (2850 kcal/kg) demonstrating the least yield, whereas those on diets of 3050, 3150, and 3250 kcal/kg showed the highest yields. Notably, carcass yield did not increase so much for dietary AMEn levels above 3050 kcal/kg, and there was no effect of sex or its interaction with AMEn on carcass yield.

It should be noted that the dietary AMEn level or the interaction between AME and sex did not affect AFP. The effect of sex on AFP was significant (*P* < 0.05). The female broilers had a higher percentage of AFP than the males (*P* < 0.05).

### Gut characteristics

The effect of the dietary AMEn level on the gut organs is presented in Table 4. The results indicated that dietary AMEn level and birds' sex had a significant interaction effect on the relative weights of the proventriculus, gizzard, and duodenum (*P* < 0.05). In other words, male and female chicks fed 2850 kcal/kg diets had the highest relative weights of proventriculus, gizzard, and duodenum. Whereas, chicks fed 3050 and 3250 kcal/kg diets had a lower relative weight of proventriculus and gizzard (*P* < 0.05). The effect of dietary AMEn level on the relative liver weight was significant, with chicks fed 2950 kcal/kg diets showing the lowest relative liver weights, while those fed 3250 kcal/kg diets exhibited the highest (*P* < 0.05). Moreover, the effect of dietary AMEn level on the relative weight of jejunum, ileum, and cecum and relative length of duodenum, jejunum, ileum, and cecum was significant (*P* < 0.05). In brief, birds fed 2850 kcal/kg diets had the highest relative jejunum, ileum, and cecum weights and relative duodenum, jejunum, ileum, and cecum length among other treatments (*P* < 0.05). It is recalled that the effect of sex on the relative length of the jejunum was significant (*P* < 0.05). In fact, female birds had the highest relative jejunum length than males (*P* < 0.05).

**Table 4:** The effect of dietary AMEn level (kcal/kg) and sex on the gut organs of broiler chicks at 43d.

Item	AMEn	Sex	**Relative weight** (Organ weight, g/ BW, g)							**Relative length** (Organ length, cm/ BW, g)			
			Proventriculus	Gizzard	Liver	Duodenum	Jejunum	Ileum	Cecum	Duodenum	Jejunum	Ileum	Cecum
AMEn × Sex	2850	Male	0.43^a^	1.49^a^	2.20	0.82^ab^	1.38	1.12	0.60	2.31	5.87	5.88	1.29
	2950	Male	0.26^c^	1.06^bc^	2.10	0.75^ab^	1.25	1.02	0.45	1.47	4.04	4.13	0.93
	3050	Male	0.22^c^	0.79^c^	2.19	0.57^ab^	1.18	1.00	0.48	0.88	2.68	2.62	0.64
	3150	Male	0.28^bc^	0.93^bc^	2.38	0.71^ab^	1.25	1.07	0.56	1.16	3.11	3.35	1.01
	3250	Male	0.24^c^	0.81^c^	2.65	0.65^ab^	1.27	0.96	0.47	1.08	3.00	3.15	0.67
	2850	Female	0.39^ab^	1.57^a^	2.40	0.86^a^	1.50	1.15	0.62	2.22	6.16	6.15	1.31
	2950	Female	0.30^bc^	1.24^ab^	2.14	0.56^b^	1.31	1.18	0.44	1.27	4.21	4.22	0.94
	3050	Female	0.22^c^	0.84^c^	2.13	0.65^ab^	1.14	0.79	0.43	1.38	2.68	2.32	0.65
	3150	Female	0.27^c^	0.88^bc^	2.36	0.70 ab	1.24	0.94	0.60	1.17	3.41	3.40	0.81
	3250	Female	0.24^c^	0.84^c^	2.41	0.64^ab^	1.18	0.84	0.56	1.15	3.08	3.26	0.71
SEM			0.024	0.07	0.12	0.064	0.07	0.08	0.05	0.14	0.12	0.18	0.08
AMEn	2850		0.41^a^	1.53^a^	2.30^ab^	0.84^a^	1.44^a^	1.13^a^	0.61^a^	2.27^a^	6.01^a^	6.01^a^	1.30^a^
	2950		0.28^b^	1.15^b^	2.12^b^	0.66^b^	1.28^ab^	1.10^ab^	0.45^b^	1.37^b^	4.12^b^	4.18^b^	0.93^b^
	3050		0.27^b^	0.81^c^	2.16^b^	0.61^b^	1.16^b^	0.89^b^	0.45^b^	1.13^b^	2.68^d^	2.47^d^	0.64^c^
	3150		0.24^b^	0.91^c^	2.37^ab^	0.70^ab^	1.24^ab^	1.00^ab^	0.58^ab^	1.16^b^	3.26^c^	3.37^c^	0.91^b^
	3250		0.22^b^	0.82^c^	2.53^a^	0.64^b^	1.23^ab^	0.90^ab^	0.52^ab^	1.11^b^	3.04^c^	3.21^c^	0.69^bc^
SEM			0.017	0.05	0.08	0.045	0.05	0.05	0.03	0.10	0.08	0.12	0.06
Sex		Male	0.29	1.02	2.30	0.70	1.27	1.03	0.51	1.38	3.74^b^	3.83	0.91
		Female	0.28	1.07	2.29	0.68	1.27	0.98	0.53	1.44	3.91^a^	3.87	0.88
SEM			0.011	0.03	0.05	0.028	0.03	0.03	0.02	0.06	0.05	0.08	0.04
P-value													
AMEn			0.0001	0.0001	0.01	0.009	0.01	0.01	0.009	0.0001	0.0001	0.0001	0.0001
Sex			0.81	0.26	0.81	0.72	0.84	0.30	0.60	0.52	0.02	0.71	0.64
AMEn × Sex			0.0001	0.0001	0.52	0.03	0.70	0.17	0.73	0.12	0.65	0.61	0.62
AMEn level													
Linear			0.0001	0.0001	0.019	0.03	0.01	0.005	0.69	0.0001	0.0001	0.0001	0.0001
Quadratic			0.0001	0.0001	0.003	0.009	0.002	0.01	0.10	0.0001	0.0001	0.0001	0.0001

a-c Means in the same row with no superscript letters or a common superscript letter after them are not significantly different (*P* > 0.05). n: 10 birds/treatment, SEM; standard error of the mean.

### Physical pellet quality

The effect of dietary AMEn level on PPQ has been reported in Table 5. Dietary AMEn level significantly influenced the starter and finisher PDI values (*P* < 0.05) but had no significant effect in the PH of either diet phase. In the starter phase, the diet containing 2850 kcal/kg (containing the 4.6 % ether extract) exhibited the highest PDI, whereas the diet with 3250 kcal/kg (containing the 6.2 % ether extract, Table 1) had the lowest PDI. In the finisher phase, the diet with 3050 kcal/kg (containing the lowest ether extract and highest crude fiber, Table 1) achieved the highest PDI, while diets contain 2850 and 3250 kcal/kg (both with the highest ether extract, Table 1) had the lowest PDI.

**Table 5:** The effect of dietary AMEn level (kcal/kg) on the PPQ of starter and finisher diets.

**AMEn**	**Starter**		**Finisher**	
	**PDI (%)**	**PH (kg)**	**PDI (%)**	**PH (kg)**
2850	90.2^a^	2.15	68.3^d^	2.60
2950	82.5^c^	1.40	70.0^c^	2.60
3050	85.7^b^	1.85	79.0^a^	2.85
3150	81.5^d^	1.70	73.9^b^	2.75
3250	65.9^e^	1.35	69.0^d^	2.65
SEM	0.12	0.14	0.28	0.46
P-value	0.0001	0.06	0.0001	0.99
AMEn level				
Linear	0.002	0.09	0.75	0.83
Quadratic	0.001	0.26	0.04	0.91

a-e Means in the same row with no superscript letters or a common superscript letter after them are not significantly different (*P* > 0.05). PDI; pellet durability index (*n* = 4 replicates), PH; pellet hardness (*n* = 10 replicates), SEM; standard error of the mean.

## Discussion

### Growth performance and physical pellet quality

In the present study, a significant interaction between dietary AMEn level and sex was observed for BW, FI, FCR, and EPEF (Table 2). The male and female growth performance response to dietary AMEn followed a quadratic pattern, with the best results achieved by 3050 kcal/kg diets (Table 2 and Fig. 1, Fig. 2, Fig. 3). Moreover, the birds fed 3050 kcal/kg diets had higher finisher PDI, which confirmed Svihus' (2006) statement that the macrostructure of pellet measured by PDI can alter FI and BW of broilers. The birds consuming a 2850 kcal/kg diet exhibited a higher starter PDI compared to other treatments. However, this increased starter PDI was deemed less significant due to the higher feed intake observed in the finisher period relative to the starter period. Therefore, the magnitude of PPQ, namely PDI values, is much greater for finisher diets than starter diets (Mohammadi Ghasem Abadi et al., 2019a).

The quadratic relationship observed for FI and, consequently, BW (Fig. 1, Fig. 2) can be explained by gut filling in birds fed low-energy diets and the impaired finisher PDI resulting from higher MAF inclusion, which increased the ether extract content of the feed. Specifically, the physical capacity of the gut is the limiting factor in low AMEn diets, while reduced PPQ (lower PDI) in high AMEn diets serves as an upper limiting factor, affecting FI (Lopez and Leeson, 2008). Limitations in gut capacity, particularly during the early rearing period, play a major role in reducing growth performance in birds fed low AMEn diets, and this effect diminishes with age (Hidalgo et al., 2004; Ravindran and Abdollahi, 2021). Conversely, increasing MAF in high AMEn diets linearly decreases PDI (Mohammadi Ghasem Abadi et al., 2019b; Massuquetto et al., 2020). Furthermore, increasing dietary AMEn did not produce a linear or quadratic response in PH of either starter or finisher diets. In other words, PH remained unchanged even when PDI decreased quadratically with higher AMEn levels.

These data are inconsistent with the findings of Abdollahi et al. (2018), who reported a decrease in FI from 1 to 21 days with increasing dietary AMEn. However, broiler growth responses to dietary AMEn have been inconsistent. Abdollahi et al. (2018) observed an interaction between dietary AMEn density and feed form (mash vs. pellet), whereas Massuquetto et al. (2020) found no such interaction. In the present study, broilers of both sexes fed high-energy pellet diets (>3050 kcal/kg) exhibited similar growth performance, which was lower than that of birds fed 3050 kcal/kg diets. This result aligns with Massuquetto et al. (2020), who reported that increasing AMEn density of pellet diets above 3121 kcal/kg from 35 to 47 days had no significant effect on FI, BW, or FCR.

Contradictory results have been reported regarding dietary AMEn level on the growth performance of male and female broiler chicks (Maynard et al., 2019). This finding showed that male broilers had higher FI and BW than the female broilers, consistent with previous research (Ojedapo et al., 2008; Lopez et al., 2011; Benyi et al., 2015; Shim et al., 2012). The present study offers valuable insights into the growth performance of broiler chickens. It was found that there is a linear relationship between dietary AMEn and growth performance; however, the quadratic models for growth performance were preferred over the linear models because they exhibited a higher R² value. This suggests that the factors influencing the growth performance of broilers fed a pellet are likely more complex than previously recognized. Additionally, by employing these growth performance equations in the nonlinear programming software proposed by Guevara (2004), we can greatly enhance the optimization of performance responses to energy density.

### Carcass traits

The effects of dietary AMEn levels on carcass characteristics, particularly the AFP percentage, have been inconsistent in previous studies (Clark, 2015). In the present study, higher AMEn diets (>2950 kcal/kg) resulted in similar carcass yields. This study confirmed that in both sexes, the best results were achieved by birds fed 3050 kcal/kg diets for carcass and breast yield. Conversely, birds fed 2850 kcal/kg diets exhibited the lowest carcass and breast yields, while showing the highest relative weights of back and wings compared to other treatments.

It should be noted that by increasing AMEn level, the leg and AFP percentages did not lead to a linear or quadratic increase. However, the sex effect significantly influenced the AFP percentage. Females exhibited a higher AFP due to greater fat deposition compared to males, aligning with findings by Madilindi et al. (2018) and Maynard et al. (2019), while Udeh et al. (2015) reported no sex differences in carcass traits. It seems that hormonal variations may explain differences in fat deposition, with higher AMEn levels promoting fat accumulation in females (Santos et al., 2005; Fouad and El-Senousey, 2014). Santos et al. (2005) stated that females exhibited higher breast, AFP yield, as confirmed by our results. However, a significant interaction between dietary AMEn and sex was observed on breast, wing, and foot yield.

In the present study, the wing yield was higher in light-weight birds (birds fed 2850 kcal/kg diets), which was confirmed by Dilizi et al. (2010). Muñoz et al. (2018) showed that males had higher carcass and leg weight but lower AFP than females. Conversely, breast and leg weights were higher in females compared to males, and these results are in line with the results of Mendes et al. (2004), Lima et al. (2008), and Lopez et al. (2011). However, Siaga et al. (2017) observed no sex effect on breast relative weight. Benyi et al. (2015) reported sex-related differences in the relative weights of back, wings, and legs, but not in breast relative weight. In the current study, the legs relative weight was not influenced by the dietary AMEn, Sex and their interaction, as confirmed by Kim et al. (2016), who did not observe any effect of dietary AMEn on the relative weight of the liver, breast, and legs.

### Gut characteristics

The dietary AMEn had a significant impact on the relative weight of the gut organ compared to the sex effect. In fact, by increasing AMEn levels, the relative weights of digestive organs changed linearly and quadratically, except for the relative weight of the cecum.

According to the results of this study, the highest and lowest relative weights of the proventriculus, gizzard, jejunum, ileum, and cecum were found in lighter birds (chicks fed 2850 kcal/kg diets) and heavier-BW (chicks fed 3050 and 3250 kcal/kg diets), respectively.

In other words, the lower the BW of the bird, the higher the relative weight of its gut organs, because the relative weight of the gut organs is calculated by dividing the absolute weight of the organ by the BW.

Saldaña et al. (2016) observed that the dietary AMEn level does not affect the relative weight of gut organs. In contrast, De Verdal et al. (2011) found a positive correlation between the dietary AMEn level and the weight of the liver and proventriculus. Enhanced development of the proventriculus and gizzard improves the grinding of digesta, secretion of hydrochloric acid and pepsinogen by the proventriculus, and facilitates reflux from the duodenum.

Recent studies have shown that the relative weight and length of the gut organ influenced by dietary AMEn, which provokes FI and ultimately BW. Moreover, De Verdal et al. (2010) indicated that intestinal adaptation occurs through changes in the relative length and weight of intestinal segments, enabling low digestive efficiency broilers to compensate for gastric area functionality.

Lamot et al. (2019) found that increased diet energy density raises FI, affecting the length and weight of the gut organs. Variations in FI and nutrient intake contribute to small intestine growth. Reduced FI may decrease digestion needs, while higher nutrient density promotes intestinal development.

However, Abdollahi et al. (2018) report no effect of dietary AMEn on the relative weight of the duodenum, jejunum, and ileum. Huang et al. (2022) found that there was a negative correlation between growth performance and gizzard weight, indicating that a larger gizzard was significantly associated with a better FCR, while there was a positive correlation between growth performance and relative liver weight. However, our data illustrated that as the dietary AMEn level increased, the gizzard relative weight decreased, which was in agreement with Abdollahi et al.'s (2018) results. In general, Svihus (2006) pointed out that the microstructure of the pellet, namely, feed particle size, can stimulate gizzard growth development and untimely changes in nutrient digestibility. It seems that as the dietary AMEn increased, the percentage of corn reduced, as the only altering feed texture factor decreased feed particle size, which can cause a lower relative weight of the gizzard. This phenomenon was observed in Abdollahi et al. (2018) results, which reported a decrease in apparent ileal digestibility of nitrogen and starch with concomitant reduction in gizzard relative weight when dietary AMEn was increased. However, Rougiere et al. (2009) found that improving feed efficiency significantly increased proventriculus and gizzard absolute weight and decreased small intestine weight in chicks. The liver weight of low-feed-efficiency chicks may compensate for deficiencies in gizzard digestion by improving digestion and absorption in the small intestine (Rideau et al., 2014). Lamot et al. (2019) observed an increase in the relative weight of the liver with increasing diet density. De Verdal et al. (2011) found that there was a positive correlation between the relative weight of the liver and the proventriculus and the diet density. They also showed that there was a negative correlation between the dietary energy level and the relative weight of the gizzard, proventriculus, and liver.

Garcia et al. (2007) and Hetland et al. (2001) observed a negative correlation between growth performance and the relative length of the cecum. A longer cecum may enhance feed efficiency by increasing the duration of chyme fermentation and digestion by cecal microbiota (Huang et al., 2022).

The present study demonstrated that both dietary AMEn and sex had a significant impact on the relative length of the jejunum. The linear and quadratic reduction in the relative lengths of different segments of the small intestine in response to increased dietary AMEn level warrant further investigation to elucidate the underlying mechanisms.

## Conclusion

Therefore, our findings showed that the bird's growth performance is a result of dietary AMEn density and PPQ. In other words, there is an optimum dietary AMEn value in broiler-fed pellet diets. Routinely, poultry nutritionist reduces the dietary AMEn to provide the least-cost feed. Selection of an inappropriate dietary AMEn level not only can alter the performance but also has a great impact on broiler carcass and gut characteristics. The optimal dietary energy level identified in this study for Hubbard Flex broiler-fed pellet diets is 3050 Kcal/kg. Therefore, these simple quadratic equations of growth performance will help nutritionists to choose the best dietary AMEn level to achieve maximum overall growth performance from 1 to 42 days. However, further studies were needed to present complex growth performance models that include AMEn and age parameters for dynamic feed formulation in each age based on nonlinear feed formulation software.

## Disclosures

The author declare that they have no conflict of interest with respect to the author or publication of this article " Effect of apparent metabolizable energy and sex on predictive models for growth performance, carcass traits, gut characteristics, and pellet quality in broiler chickens"

## CRediT authorship contribution statement

**Mohammad Hossein Mohammadi:** Writing – original draft, Supervision, Resources, Methodology, Investigation, Funding acquisition, Formal analysis, Conceptualization. **Mahya Pahlavan Hassan:** Writing – review & editing, Software, Resources, Data curation. **Enayat Rahmatnejad:** Writing – review & editing, Validation, Software, Methodology, Formal analysis.
